# Prevalence of Vancomycin-Resistant *Enterococcus* (VRE) in Poultry in Malaysia: The First Meta-Analysis and Systematic Review

**DOI:** 10.3390/antibiotics11020171

**Published:** 2022-01-28

**Authors:** Yusuf Wada, Ahmad A. Irekeola, Rafidah H. Shueb, Mustapha Wada, Hafeez A. Afolabi, Chan Y. Yean, Azian Harun, Abdul R. Zaidah

**Affiliations:** 1 Department of Medical Microbiology and Parasitology, School of Medical Sciences, Universiti Sains Malaysia, Kubang Kerian 16150, Malaysia; wadayusuf34@gmail.com (Y.W.); irekeola@student.usm.my (A.A.I.); hanimkk@usm.my (R.H.S.); yychan@usm.my (C.Y.Y.); azian@usm.my (A.H.); 2 Department of Zoology, Faculty of Life Sciences, Ahmadu Bello University, Zaria 810211, Nigeria; 3 Microbiology Unit, Department of Biological Sciences, College of Natural and Applied Sciences, Summit University Offa, Offa PMB 4412, Nigeria; 4 Faculty of Veterinary Medicine, Ahmadu Bello University, Zaria 810211, Nigeria; musty.wada@gmail.com; 5 Department of General Surgery, School of Medical Sciences, Universiti Sains Malaysia, Kubang Kerian 16150, Malaysia; Hafeez11@student.usm.my; 6 Hospital Universiti Sains Malaysia, Universiti Sains Malaysia, Kubang Kerian 16150, Malaysia

**Keywords:** vancomycin-resistant *Enterococcus*, meta-analysis, poultry, Malaysia

## Abstract

Databases such as PubMed, Scopus and Google Scholar were searched. Data extraction and assessment of study protocol was done by two independent reviewers and the results were reviewed by a third. OpenMeta analyst and comprehensive meta-analysis (CMA) were used for the meta-analysis. The random effect model was used, publication bias and between-study heterogeneity was assessed. Seventeen studies were added to the final meta-analysis. Studies were sampled from 2000–2018 and of the 8684 isolates tested, 2824 were VRE. The pooled prevalence of VRE among poultry in Malaysia was estimated at 24.0% (95% CI; 16.7–33.1%; *I*^2^ = 98.14%; *p* < 0.001). Between-study variability was high (*t*^2^ = 0.788; heterogeneity *I*^2^ = 98.14% with heterogeneity chi-square (*Q*) = 858.379, degrees of freedom (df) = 16, and *p* < 0.001). The funnel plot showed bias which was confirmed by Egger’s test and estimates from the leave-one-out forest plot did not affect the pooled prevalence. Pooled prevalence of VRE in chickens and ducks were 29.2% (CI = 18.8–42.5%) and 11.2%, CI = 9.0–14.0%) respectively. *Enterococcus faecalis* was reported most with more studies being reported in Peninsular Malaysia Central region and used antibiotic disc diffusion as detection method. Increased surveillance of VRE in poultry in Malaysia is required.

## 1. Introduction

*Enterococcus* has emerged as a significant nosocomial and community-acquired pathogen as a result of its ability to develop resistance to antimicrobials, particularly vancomycin. Vancomycin is the final treatment option, particularly for *Enterococcus* [1,2]. Human antimicrobial use, as well as their use as growth promoters in the livestock industry, were thought to have resulted in the emergence of enterococcal-resistant strains. A good example is the use of avoparcin as a feed additive to promote livestock growth [3].

The National Pharmaceuticals Regulatory Agency (NPRA) and the Department of Veterinary Services (DVS) in Malaysia have prohibited the use of avoparcin and vancomycin to reduce the spread or prevalence of vancomycin-resistant *Enterococcus* (VRE). DVS has been monitoring veterinary drug residues, including antibiotics, in animal feed since 2013, in accordance with EEC Directive 1990 [4]. This will invariably entail the monitoring of two antibiotic groups: group A, which includes banned substances such as avoparcin, chloramphenicol and vancomycin, and group B, which includes drugs with MRLs such as tetracycline. The most frequently used antibiotic classes in Malaysia are aminoglycosides, Beta-Lactams, microlides, tetracyclines, polymyxins, quinolones, sulfonamides and amphenicols [4]. What has brought VRE to the forefront in Malaysia is not only its critical public health concern but its potential economic impact on the livestock sector [3]. Antibiotic resistance poses great threat to food safety and public health when the resistant bacteria spread from food animals to poultry farmers, farmworkers and veterinarians through the food chain. As a result, antimicrobial resistance in poultry is a significant public health risk that warrants a discreet yet robust response. VRE has been reported in Malaysia amongst health workers, animals, hospital patients and farmworkers [3]. The epidemiology and transmission of resistant bacteria between humans and animals has increased, and their zoonotic potential cannot be underestimated [5].

In order to assess the risks and distribution of vancomycin-resistant *Enterococcus* (VRE) in poultry in Malaysia, a meta-analysis and a systematic review were carried out. This could help provide basic information for vigilance and the conceptualization of suitable and tailored policies in Malaysia to control antimicrobial resistance in poultry.

## 2. Results

### 2.1. Search Results and Eligible Studies

**Figure 1:**
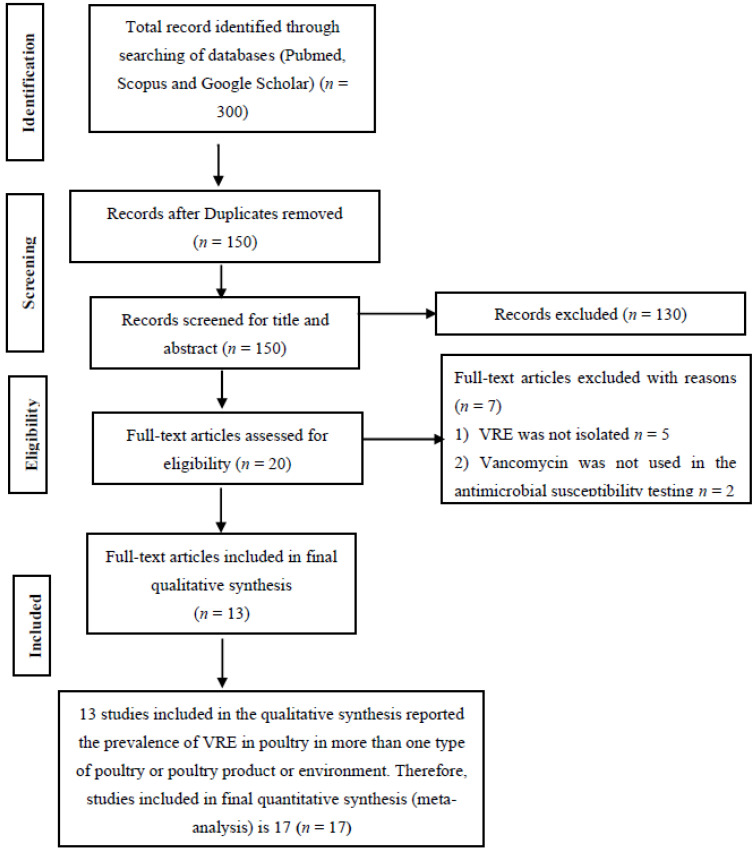
PRISMA flow diagram for the selection of eligible articles included in the study.

A total of 300 studies were identified through searching of databases and 150 duplicates were removed. The 150 articles left had their titles and abstracts screened, and 130 articles were excluded having found not to meet any of the inclusion criteria. Twenty full-text articles were assessed for eligibility with seven excluded for lack of sufficient information and the non-use of vancomycin for the antimicrobial susceptibility test (Figure 1). A total of 13 full-text studies were used for qualitative analysis (Figure 1). To have a near accurate estimate of VRE in poultry in Malaysia, studies reporting the prevalence in more than one type of poultry bird, environment or poultry product were analyzed as different studies. Ten studies reported the prevalence in a single poultry bird, poultry environment or poultry product while three studies reported the prevalence in more than one [6,7,8] (Table 1).

### 2.2. The Pooled Prevalence of VRE in Poultry in Malaysia

**Figure 2:**
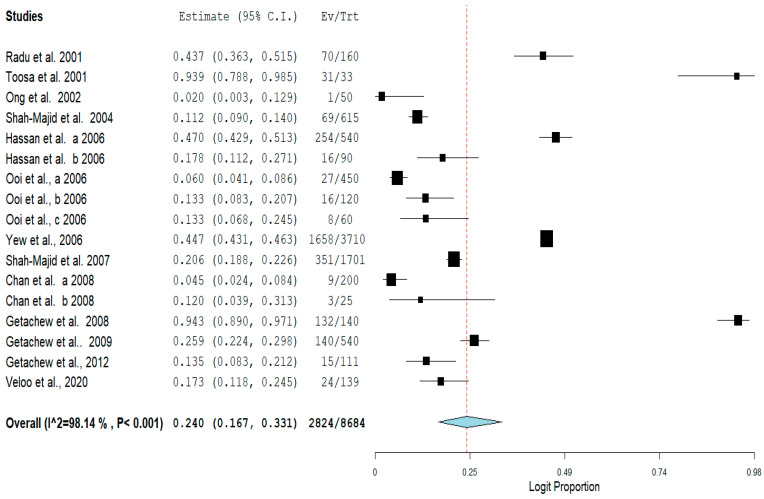
Forest plot showing the pooled prevalence of VRE in poultry in Malaysia.

**Figure 3:**
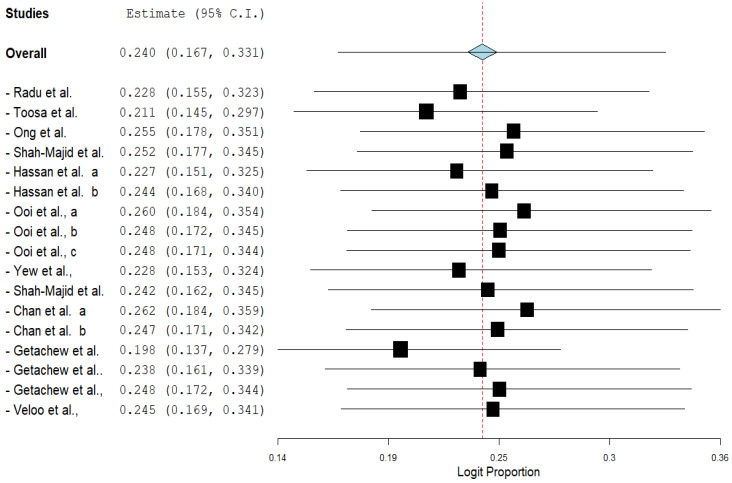
Leave-one-out forest plot of VRE in poultry in Malaysia.

**Figure 4:**
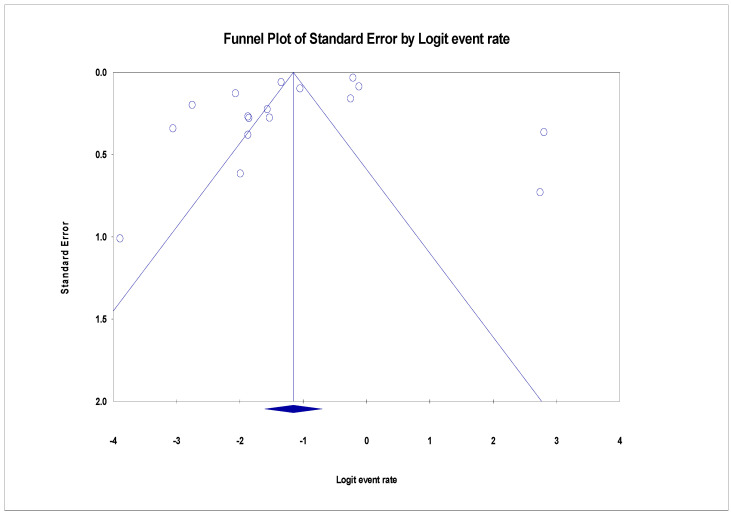
Funnel plot showing publication bias in studies reporting the prevalence of VRE in in poultry in Malaysia. Studies on the right side are fewer than those on the left and thus asymmetrical. The funnel plot is used for the visualization of bias.

**Figure 5:**
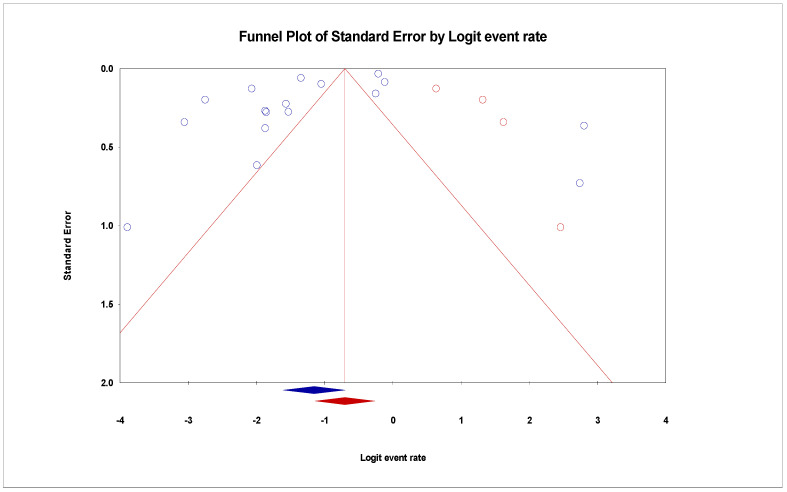
Funnel plot showing 4 added studies (in Red) in the Trim-and-Fill method reporting the prevalence of VRE in in poultry in Malaysia. This method simply looks for missing studies that will eventually eliminate bias. In this case, 4 studies will have to be added to the right side for the plot to be symmetrical.

The pooled prevalence of VRE in poultry in Malaysia was estimated at 24.0% (95% CI; 16.7–33.1%; *I*^2^ = 98.14%; *p* < 0.001) (Figure 2). Random-effects meta-analyses were carried out using the total sample size and number of positives (effect size, standard error of effect size) to estimate the prevalence of VRE in poultry in Malaysia. Between-study variability was high (*t*^2^ = 0.788; heterogeneity *I*^2^ = 98.14% with heterogeneity chi-square (*Q*) = 858.379, degrees of freedom (df) = 16, and *p* < 0.001). No individual study affected the heterogeneity and pooled prevalence of VRE in poultry in Malaysia as seen in the leave-one-out forest plot that was generated in the sensitivity analysis (Figure 3). More so, publication bias was observed as shown in the asymmetrical funnel plot (Figure 4). In addition to the funnel plots, the Trim-and-Fill method was then applied to include the “missing” studies from the analysis. The asymmetric studies were trimmed to locate the unbiased effect and fills the plot by re-inserting the trimmed studies as well as their imputed counterparts. Accordingly, four studies were missed and fell at the right side of the pooled estimate (Figure 5). In the Trim-and-Fill method, the adjusted estimate of VRE in poultry in Malaysia was 33.12% (95% CI; 24.3–43.3%). The Egger’s regression *t* = 1.777 (intercept = −4.0972; 95% CI; −0.75–2.57; *p* = 0.096) and Begg’s rank test (*p* = 0.30310) did not suggest significant publication bias.

### 2.3. Subgroup Meta-Analysis

To identify the possible sources of heterogeneity among studies, as substantial heterogeneity was observed, subgroup analysis was carried out using the year of study of the included studies, the regions where the studies were reported, the sources of VRE isolates and the method used in detecting VRE.

**Figure 6:**
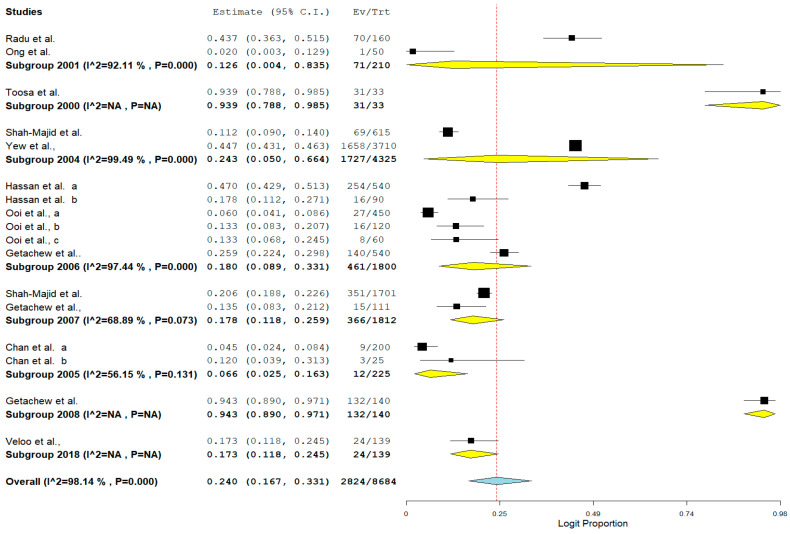
Forest plot showing the subgroup meta-analysis by study year of VRE in poultry in Malaysia.

The result of subgroup meta-analysis by study year revealed overall large variability in studies reporting the prevalence of VRE (the Higgins *I*^2^ statistic = 98.14% with heterogeneity chi-square (*Q*) = 858.379, degrees of freedom = 16, and *p* < 0.001). However, most studies (*n* = 6) were reported in 2006 with only a single study reported as recently as 2018 and published in 2020 (Table 2). Studies carried out in 2005 (*n* = 2) and 2004 (*n* = 2) had a moderate (*I*^2^ = 56.15%) and highest (*I*^2^ = 99.49%) heterogeneity respectively (Table 2). The forest plot for subgroup meta-analysis by study year is also shown in Figure 6.

**Figure 7:**
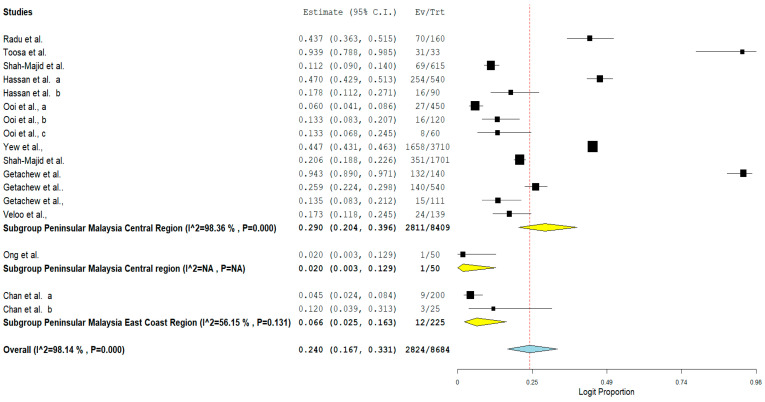
Forest plot showing the subgroup meta-analysis by study region of VRE in poultry in Malaysia.

The result of subgroup meta-analysis by study region showed that majority of the studies (*n* = 14) that reported the prevalence of VRE in poultry in Malaysia were from the Central region of Peninsular Malaysia with a prevalence of 29% and a high heterogeneity (*I*^2^ = 98.36%). The East coast region of the Malaysian Peninsular only had 2 studies with a prevalence of 6.6% and a moderate heterogeneity (*I*^2^ = 56.15%) (Table 3). In addition, the forest plot of subgroup meta-analysis by study region is shown in Figure 7.

**Figure 8:**
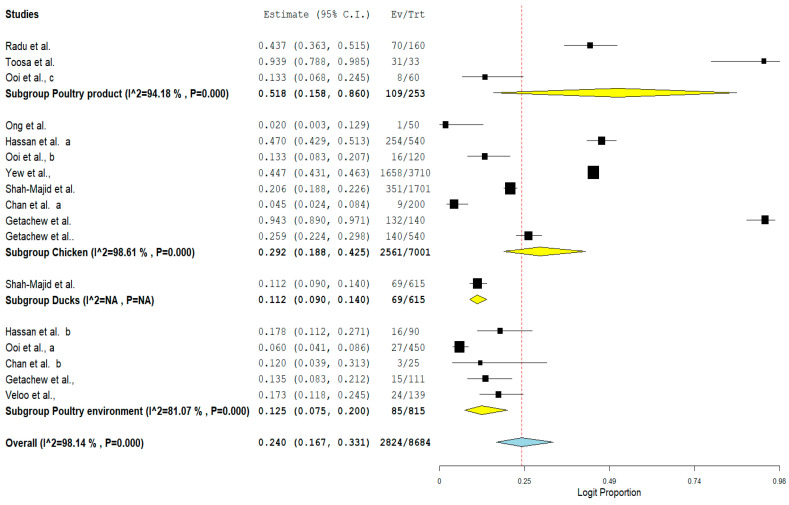
Forest plot showing the subgroup meta-analysis by Isolate sources of VRE in poultry in Malaysia.

Furthermore, the result of subgroup meta-analysis according to isolate sources revealed that most of the studies (*n* = 8) had their VRE isolated from chicken with a prevalence of 29.2% and a high heterogeneity (*I*^2^ = 98.61%) (Table 4). This was followed by 5 studies reporting the isolation of VRE from poultry environment with a prevalence of 12.5% and also a high heterogeneity (*I*^2^ = 81.07%) (Table 4). The forest plot is also shown in Figure 8.

**Figure 9:**
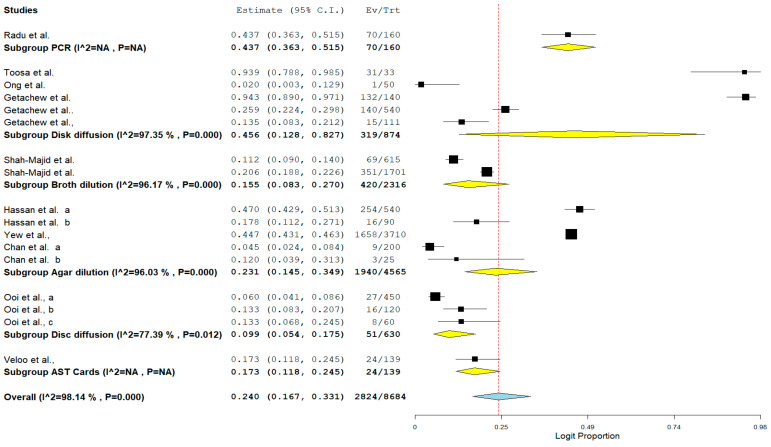
Forest plot showing the subgroup meta-analysis by detection method of VRE in poultry in Malaysia.

Lastly, the result of subgroup meta-analysis according to the detection method showed that 5 studies each utilized disc diffusion and agar dilution with a prevalence of 45.6% and 23.1%, respectively (Table 5). Only one study utilized polymerase chain reaction (PCR) as a method of detection with a prevalence of 43.7% and CI of 36.3–51.5%. (Table 5). The forest plot is shown in Figure 9.

### 2.4. Meta-Regression

A separate meta-analysis was performed for each variable included and these are: the study year; the study region; the isolate source; and the detection method. A multivariate meta-regression analysis was utilized when the *p*-values of the variables in a single meta-regression is <0.25. In the final meta-analysis, all the variables listed were included. In the multivariate meta-regression, no analysis was recorded for studies carried out in 2007, 2008 and 2018. Most of the variables analyzed using multivariate meta-regression contributed to the heterogeneity observed in this study with a *p*-value of <0.05. Exceptions to these were studies whose isolates were from poultry products (*p* = 0.0873) and chicken (*p* = 0.520), study that utilized the use of AST cards as its detection methods (*p* = 0.427) and a study carried out in the year 2000 (*p* = 0.994) (Table 6).

### 2.5. Species Distribution of Enterococcus in Poultry in Malaysia

Nine species of *Enterococcus* were isolated in 11 studies reporting the prevalence of VRE in poultry in Malaysia, while 2 studies [13,18] did not report the species of *Enterococcus* isolated. *Enterococcus faecalis* was the most isolated (*n* = 563) and this was followed by *E. faecium* with 201 isolates (Table 7).

## 3. Discussion

This is the first study to use meta-analysis and a systematic review to determine the prevalence of VRE in poultry in Malaysia, to the best of our knowledge. The pooled prevalence in this study is based on a thorough analysis of data from scientific publications on the prevalence of VRE in poultry in Malaysia published between 2001 and 2020. A meta-analysis was performed on 17 studies. The literature reviewed was heterogeneous, as expected, because the review included VRE reports from various regions, different study years, a variety of isolate sources and different methods for VRE detection. As a result, a random effect size model was used. This study’s high heterogeneity could be attributed to small-study effects and publication bias, because smaller studies sometimes show unusual, and often larger, treatment effects when compared to larger ones.

A small study with a larger-than-average impact is more likely to meet the statistical significance criterion, which may lead to an overestimation of true therapeutic effects. The assessment of publication bias is critical in meta-analysis. This is due to the fact that not all research findings are published, particularly those that are deemed unfavorable to a developed protocol or product, or those that would elicit only a minor amount of interest. Thus, studies that report relatively significant treatment effects are more likely to be submitted and/or approved for publication than studies that report more modest treatment effects. Our meta-analysis revealed a high level of variability, implying that the observed variability was compensated for by factors other than chance. The majority of the variables examined in these studies resulted in the observed heterogeneity. Similarly, studies with isolates from poultry products and chicken, studies with AST cards as detection methods and a study conducted in the year 2000 were not indicators of study heterogeneity.

There were no previous studies on meta-analysis to compare the prevalence of VRE in poultry in Malaysia, as this is the first study to analyze the prevalence of VRE in poultry in Malaysia. However, Wada et al. [1] reported a VRE pooled prevalence of 25% in Malaysia. In this current review, studies reporting the highest prevalence of VRE in poultry in Malaysia were mostly carried out in the Central region of the country. Most universities and research facilities such as the Department of Veterinary Services located in Putrajaya and National Pharmaceuticals Regulatory Agency (NPRA) which specializes in the surveillance of pathogens and regulations of drugs are located in the Central region of the country and this could be the reason why most of the studies were reported from that region. The density of poultry production in these regions could also be a factor. It is important that other regions are actively involved in the surveillance of *Enterococcus* and other pathogens, as this will help project the true estimate of the prevalence of VRE in poultry in Malaysia. Further, more up-to-date research is required as the most recent study reporting the prevalence of VRE in poultry was carried out in 2018 and published in 2020. It is either because the occurrence of resistant *Enterococcus* has reduced as a result of the ban on avoparcin or there is simply not enough surveillance going on to ascertain an actual estimate. Avoparcin and vancomycin were banned in Malaysia since 2013 by the National Pharmaceuticals Regulatory Agency (NPRA) and the Department of Veterinary Services (DVS) [4].

It is not surprising that most of the studies reported the isolation of VRE from chickens. Malaysians were expected to consume 48.7 kg of poultry meat per person in 2021, according to projections. This places Malaysia among the world’s top consumers of poultry meat [4]. In addition, the disc diffusion and agar dilution were the two most utilized VRE detection methods in poultry in Malaysia. Eight studies utilized the disc diffusion method, which has been reported to be straightforward and functional, with a well-standardized design, and it has the ability to provide categorical data that are simply understood by all practitioners, as well as the ability to choose from a variety of discs to test [19]. The lack of mechanization or automation of the disc test is one of its drawbacks. Only one study utilized PCR in the detection of VRE. For the detection and characterization of distinct VRE species, PCR is a quicker and more sensitive approach [20]. Finally, *E. faecalis* was the most isolated species of VRE in poultry in Malaysia from our analysis. The most frequent species capable of producing illness and creating an antibiotic resistance concern are *E. faecalis* and *E. faecium*, with *E. faecalis* accounting for the bulk of infections [21] *E. faecalis* is now recognized as a severe source of both hospital- and community-acquired urinary tract infections (UTIs), which can result in serious, life-threatening consequences such as bacteremia [22].

The use of antibiotics in animal husbandry varies by location and country. Antibiotics are sold in significantly larger quantities in low- and middle-income nations than they are in high-income countries [23]. As a region of fast-developing and integrated economies, Southeast Asia (SEA) is considered a hotspot location for antimicrobial resistance (AMR) [24]. Low- and middle-income nations utilized more antibiotics classified as medically important when compared to high-income countries [23]. The presence of veterinary drug traces in food samples confirmed this, indicating that violation rates in underdeveloped nations were higher than in developed nations [25].

SEA countries have had fast growth in the aquaculture and poultry production sectors in recent decades, accounting for a relatively large proportion of the worldwide veterinary antibiotic market [24]. Antibiotic management and therapy has become one of the most effective ways for these countries to avoid uncontrolled epidemic infections that could threaten their economies [24].

## 4. Materials and Methods

### 4.1. Study Design and Protocol

For this study, the Preferred Reporting Items for Systematic Reviews and Meta-Analysis Protocol (PRISMA-P 2015) guidelines [26] was used as the checklist (Supplementary File S1).

### 4.2. Literature Review

Abstracts from PROSPERO database and database of abstracts of reviews of effects (DARE) (http://www.library.UCSF.edu (accessed on 5 November 2021) were searched to ensure that no other meta-analysis on the prevalence of VRE in poultry in Malaysia exists or is ongoing. This was then followed by searching PubMed, Scopus and Google Scholar for published studies about the prevalence of VRE in poultry in Malaysia. These databases were searched using the search strategy; (“vancomycin resistant enterococci”[MeSH Terms] OR (“vancomycin resistant”[All Fields] AND “enterococci”[All Fields]) OR “vancomycin resistant enterococci”[All Fields] OR (“vancomycin”[All Fields] AND “resistant”[All Fields] AND “enterococcus”[All Fields]) OR “vancomycin resistant enterococcus”[All Fields] OR “VRE”[All Fields] OR (“vancomycin resistant enterococci”[MeSH Terms] OR (“vancomycin resistant”[All Fields] AND “enterococci”[All Fields]) OR “vancomycin resistant enterococci”[All Fields] OR (“vancomycin”[All Fields] AND “resistant”[All Fields] AND “enterococcus”[All Fields]) OR “vancomycin resistant enterococcus”[All Fields])) AND (“poultry”[MeSH Terms] OR “poultry”[All Fields] OR “poultries”[All Fields] OR “poultry s”[All Fields]) AND (“malaysia”[MeSH Terms] OR “malaysia”[All Fields] OR “malaysia”[All Fields]). In addition, references and titles from included articles were utilized as a supplementary search tool. Two authors carried out the search to minimize bias.

### 4.3. Inclusion and Exclusion Criteria for Studies

All cross-sectional or cohort studies that reported the prevalence of VRE isolates or numbers of VRE and total enterococci isolates in poultry, poultry environment and poultry products in Malaysia were included. In addition, studies published or reported in English in which the standard method (method approved for use according to the Clinical and Laboratory Standards Institute (CLSI) and other guidelines) was used to detect VRE were included. Studies with insufficient information, studies on antimicrobial susceptibility tests other than vancomycin, studies not reporting enterococcal isolates separately (no population denominator), reviews, comments and duplications, case report studies and studies that did not report the prevalence of VRE in poultry in Malaysia were excluded.

### 4.4. Data Extraction

Identification of studies was conducted based on our exclusion criteria and studies to be included were analyzed according to their title, abstract and full text. The first author’s name, publication year, study year, isolate sources, study region, number of cases involved in the studies, detection method, sample size, *Enterococcus* species isolated and the prevalence of VRE in poultry were extracted from the manuscripts. Two independent reviewers extracted all data from the included articles, and the results were reviewed by a third reviewer. Discrepancies between the reviewers were resolved by a consensus.

### 4.5. Study Quality Assessment

The Joanna Briggs Institute (JBI) critical appraisal checklist for prevalence data [27] (Supplementary File S2) was used to evaluate the quality of the included studies. This appraisal checklist contains 9 items that assess: (1) appropriate sampling frame; (2) proper sampling technique; (3) adequate sample size; (4) study subject and setting description; (5) sufficient data analysis; (6) use of valid methods for the identified conditions; (7) valid measurement for all participants; (8) using appropriate statistical analysis; and (9) adequate response rate. Each item is graded as yes, no, unclear or not applicable. A score of 1 was allotted for the ‘yes’ response, while 0 scores were provided for ‘no’ and ‘unclear’ responses. Finally, the mean score was calculated for each article. Then, studies with scores below and above the mean were characterized as poor and good quality, respectively [24]. Studies were included in the analysis if consensus was reached among two reviewers. The quality of the 13 included studies is given in (Supplementary File S3).

### 4.6. Data Analysis

Prevalence of VRE in poultry in Malaysia was calculated, and subgroup analyses were performed according to the study year, study region, the isolate sources and detection method. Where the prevalence was not reported by a study, they were back-calculated. DerSimonian and Laird method of meta-analysis [28,29] was used to determine the pooled prevalence. The random-effect model was used because heterogeneity was anticipated given that the studies were carried out in diverse locations and settings.

### 4.7. Bias and Heterogeneity Analysis

Within-study biases were evaluated by the study region, study year, isolate sources, and detection method. Small study effects or bias were examined by funnel plots and trim-and-fill plots. The heterogeneities of study-level estimates were assessed by Cochran’s *Q* test. Non-significant heterogeneity was accepted if the ratio of Q and the degrees of freedom (Q/df) was less than one. The percentage of the variation in prevalence estimates attributable to heterogeneity was measured by the inverse variance index (*I*^2^), and *I*^2^ values of 25%, 50% and 75% were considered to be of low, moderate and high heterogeneity, respectively [29]. The sources of heterogeneity were analyzed using the sensitivity analysis (leave-one-out meta-analysis), subgroup analysis and meta-regression. Meta-analysis was performed using OpenMeta Analyst software [30] and Comprehensive meta-analysis version 2 [31].

## 5. Conclusions

There is abundant proof that drug-resistant bacteria exist in poultry and can be transmitted to humans. A systematic review and meta-analysis of studies reporting the prevalence of VRE in poultry in Malaysia was conducted and a pooled prevalence of 24.0% was obtained. However, given the observed relatively high heterogeneity, it is hard to conclude that this estimate reflects the real point estimate. Nonetheless, we believe the estimate gives a good idea of the prevalence of VRE in poultry in Malaysia. With the emergence of drug-resistant bacterial strains, including vancomycin, there is a need to investigate newer antimicrobials in veterinary medicine. Based on our study, regular monitoring of VRE in poultry would aid policymakers in developing effective control measures and design AMR surveillance capacity building in Malaysia. Further, livestock farmers should be educated on antibiotics resistance and trained on responsible utilization of antibiotics. Awareness on antimicrobial resistance should be raise by all stakeholders and lastly, reduction of contamination by encouraging proper hygiene in animal husbandry, food production and processing.

## Figures and Tables

**Table 1:** Characteristics of included studies reporting the prevalence of vancomycin-resistant enterococcus in poultry in Malaysia.

S/No.	Author, Publication Year	Study Year	Study Region	Isolate Sources	Sample Size	Number Positive	Prevalence (%)	Detection Method
1	[9] Radu et al., 2001	2001	Peninsular Malaysia Central Region	Poultry product	160	70	43.75	PCR
2	[10] Toosa et al., 2001	2000	Peninsular Malaysia Central Region	Poultry product	33	31	93.94	Disk diffusion
3	[11] Ong et al., 2002	2001	Peninsular Malaysia Central region	Chicken	50	1	2.00	Disk diffusion
4	[12] Shah-Majid et al., 2004	2004	Peninsular Malaysia Central Region	Ducks	615	69	11.22	Broth dilution
5a	[6] Hassan et al., 2006a	2006	Peninsular Malaysia Central Region	Chicken	540	254	47.03	Agar dilution
5b	[6] Hassan et al., 2006b	2006	Peninsular Malaysia Central Region	Poultry environment	90	16	17.78	Agar dilution
6a	[7] Ooi et al., 2006a	2006	Peninsular Malaysia Central Region	Poultry environment	450	27	6.00	Disc diffusion
6b	[7] Ooi et al., 2006b	2006	Peninsular Malaysia Central Region	Chicken	120	16	13.33	Disc diffusion
6c	[7] Ooi et al., 2006c	2006	Peninsular Malaysia Central Region	Poultry product	60	8	13.33	Disc diffusion
7	[13] Yew et al., 2006	2004	Peninsular Malaysia Central Region	Chicken	3710	1658	44.69	Agar dilution
8	[14] Shah-Majid et al., 2007	2007	Peninsular Malaysia Central Region	Chicken	1701	351	20.63	Broth dilution
9a	[8] Chan et al., 2008a	2008	Peninsular Malaysia East Coast Region	Chicken	200	9	4.5	Agar dilution
9b	[8] Chan et al., 2008b	2008	Peninsular Malaysia East Coast Region	Poultry environment	25	3	12	Agar dilution
10	[15] Getachew et al., 2008	2008	Peninsular Malaysia Central Region	Chicken	140	132	94.29	Disk diffusion
11	[16] Getachew et al., 2009	2006	Peninsular Malaysia Central Region	Chicken	540	140	25.93	Disk diffusion
12	[17] Getachew et al., 2012	2007	Peninsular Malaysia Central Region	Poultry environment	111	15	13.51	Disk diffusion
13	[18] Veloo et al., 2020	2018	Peninsular Malaysia Central Region	Poultry environment	139	24	17.26	AST Cards

**Table 2:** Subgroup analysis for comparison of VRE in poultry in Malaysia across study year.

Study Year	Number of Studies	Prevalence (%)	95% CI	*I*^2^ (%)	*Q*	Heterogeneity Test	
						DF	*p*
2001	2	12.6	0.4–83.5	92.11	12.673	1	<0.001
2000	1	93.9	78.8–98.5	-	-	-	-
2004	2	24.3	5.0–66.4	99.49	197.659	1	<0.001
2006	6	18.0	8.9–33.1	97.44	195.688	5	<0.001
2007	2	17.8	11.8–25.9	68.89	3.214	1	0.073
2005	2	6.6	2.5–16.3	56.15	2.281	1	0.131
2008	1	94.3	89.0–97.1	-	-	-	-
2018	1	17.3	11.8–24.5	-	-	-	-

**Table 3:** Subgroup analysis for comparison of VRE in poultry in Malaysia across study region.

Study Region	Number of Studies	Prevalence (%)	95% CI	*I*^2^ (%)	*Q*	Heterogeneity Test	
						DF	*p*
Peninsular Malaysia Central Region	14	29.0	20.4–39.6	98.36	791.598	13	<0.001
Peninsular Malaysia Central Region	1	2.0	0.3–12.9	-	-	-	-
Peninsular Malaysia East Coast Region	2	6.6	2.5–16.3	56.15	2.281	1	0.131

**Table 4:** Subgroup analysis for comparison of VRE in poultry in Malaysia across isolate sources.

Isolate Source	Number of Studies	Prevalence (%)	95% CI	*I*^2^ (%)	*Q*	Heterogeneity Test	
						DF	*p*
Poultry product	3	51.8	15.8–86.0	94.18	34.374	2	<0.001
Chicken	8	29.2	18.8–42.5	98.61	502.013	7	<0.001
Ducks	1	11.2	9.0–14.0	-	-	-	-
Poultry environment	5	12.5	7.5–20.0	81.07	21.136	4	<0.001

**Table 5:** Subgroup analysis for comparison of VRE in poultry in Malaysia across detection methods.

Detection Method	Number of Studies	Prevalence (%)	95% CI	*I*^2^ (%)	*Q*	Heterogeneity Test	
						DF	*p*
PCR	1	43.7	36.3–51.1	-	-	-	-
Disk diffusion	5	45.6	12.8–82.7	97.35	151.198	4	<0.001
Broth microdilution	2	15.5	8.3–27.0	96.17	26.136	1	<0.001
Agar dilution	5	23.1	14.5–34.9	96.03	100.778	4	<0.001
Disk diffusion	3	9.9	5.4–17.5	77.39	8.847	2	0.012
AST Cards	1	17.3	11.8–24.5	-	-	-	-

**Table 6:** Final multivariable meta-regression model of VRE in poultry in Malaysia.

Variable	Coefficient	95% CI	*p*-Value
**Study region**			
Peninsular Malaysia Central Region	Reference		
Peninsular Malaysia Central Region	−6.695	−8.80–−45.91	<0.001
Peninsular Malaysia East Coast Region	−6.270	−72.28–−53.12	<0.001
**Isolate sources**			
Poultry product	Reference		
Chicken	0.069	−7.80–9.19	0.873
Ducks	−0.415	−16.78–8.48	0.520
Poultry environment	−0.918	−17.38–−0.97	0.028
**Detection method**			
PCR	Reference		
Agar dilution	3.879	27.06–50.51	< 0.001
AST Cards	−0.398	−13.80–5.84	0.427
Broth microdilution	2.507	10.85–39.29	< 0.001
Disk diffusion	2.233	11.38–33.27	< 0.001
Disk diffusion	2.985	18.33–41.38	< 0.001
**Study Year**			
2001	Reference		
2000	0.007	−18.03–18.17	0.994
2004	−3.910	−46.70–−31.50	<0.001
2005	−3.853	−45.92–−31.14	<0.001
2006	−3.673	−46.59–−26.86	<0.001
**Constant**	−0.251	−5.64–0.61	0.115

**Table 7:** *Enterococcus* species distribution in poultry in Malaysia.

Author, Publication Year	*E. faecium*	*E. faecalis*	*E. gallinarum*	*E. casseliflavus*	*E. avium*	*E. flavescens*	*E. hirae*	*E. raffinosus*	*E. durans*	Total
(Radu et al., 2001)	2	41	-	4	-	-	5	-	18	70
(Toosa et al., 2001)	-	27	-	-	-	-	4	-	-	31
(Ong et al., 2002)	-	-	-	-	-	1	-	-	-	1
(Shah-Majid et al., 2004)	28	26	-	-	4	-	-	-	-	58
(Hassan et al., 2006)	62	89	5	-	3	-	-	-	111	270
(Ooi et al., 2006)	7	4	5	-	-	-	-	35	-	51
(Shah-Majid et al., 2007)	24	236	-	-	2	-	-	-	6	268
(Chan et al., 2008)	1	1	4	-	-	-	-	3	3	12
(Getachew et al., 2008)	34	68	30	-	-	-	-	-	-	132
(Getachew et al., 2009)	36	67	17	2	-	-	-	-	-	122
(Getachew et al., 2012)	7	4	4	-	-	-	-	-	-	15
**Total**	201	563	65	6	9	1	9	38	138	

## Data Availability

The datasets used and/or analyzed during the current study are included in the manuscript.

## Supplementary Materials

The following supporting information can be downloaded at: https://www.mdpi.com/article/10.3390/antibiotics11020171/s1, File S1: PRISMA-P 2015 Checklist; File S2: The Joanna Briggs Institute (JBI) critical appraisal checklist for prevalence data. File S3: The quality of the 13 included studies.
