# Supplementary material for: Prevalence of Vancomycin-Resistant Enterococcus (VRE) in Poultry in Malaysia: The First Meta-Analysis and Systematic Review
Source: Antibiotics (Basel). 2022 Jan 28;11(2):171. doi: 10.3390/antibiotics11020171 (PMC8868266; doi:10.3390/antibiotics11020171)
Supplement: Supplementary file 1 [file antibiotics-11-00171-s001.zip › JBI STUDY QUALITY S3.pdf]

SUPPLEMENTARY FILE S3: The quality of the 13 included studies

Study name		Checklist*									Overall
		1	2	3	4	5	6	7	8	9	
1	**Radu et al. (2001)**	Yes	No	Yes	Yes	Yes	Yes	Yes	Yes	Unclear	7
2	**Toosa et al. (2001)**	Yes	No	Yes	Yes	Yes	Yes	Yes	Yes	Yes	8
3	**Ong et al. (2002)**	Yes	No	Yes	Yes	Yes	Yes	Yes	Yes	Yes	8
4	**Shah-Majid et al. (2004)**	Yes	No	Yes	Yes	Yes	Yes	Yes	Yes	Yes	8
5	**Hassan et al. (2006)**	Yes	No	Yes	Yes	Yes	Yes	Yes	Yes	Yes	8
6	**Ooi et al., (2006)**	Yes	No	Yes	Yes	Yes	Yes	Yes	Yes	Yes	8
7	**Yew et al., (2006)**	Yes	No	Yes	Yes	Yes	Yes	Yes	Yes	Yes	8
8	**Shah-Majid et al. (2007)**	Yes	No	Yes	Yes	Yes	Yes	Yes	Yes	Yes	8
9	**Chan et al. (2008)**	Yes	No	Yes	Yes	Yes	Yes	Yes	Yes	Yes	8
10	**Getachew et al. (2008)**	Yes	No	Yes	Yes	Yes	Yes	Yes	Yes	Yes	8
11	**Getachew et al. (2009)**	Yes	No	Yes	Yes	Yes	Yes	Yes	Yes	Yes	8
12	**Getachew et al. (2012)**	Yes	No	Yes	Yes	Yes	Yes	Yes	Yes	Yes	8
13	**Veloo et al., (2020)**	Yes	No	Yes	Yes	Yes	Yes	Yes	Yes	Yes	8

*** 1.** Appropriate sampling frame to address target population, **2.** Appropriate sampling way of study participants, **3.** Adequate sample size, **4.** Detail description of study participants and settings, **5.** Data analysis with sufficient coverage of identified sample, **6.** Use of valid methods to identify the condition, **7.** Standard, reliable way of measurement of condition for all participants, **8.** Availability of appropriate statistical analysis, **9.** Adequate response rate and management of low response rate

Quality of included studies by JBI critical appraisal checklist for studies reporting prevalence data
